# Clay as a Therapeutic Agent

**Published:** 1875-01

**Authors:** A. L. Knight

**Affiliations:** West Columbia, W. Va.


### CLAY AS A THERAPEUTIC AGENT.

BY A. L. KNIGHT, M. D., OF WEST COLUMBIA, W. VA.

[*Read before the Meigs County (Ohio) Medical Society.*]

Clay as a remedial agent is comparatively new; in fact, if not entirely new, it at least has been but a few years in use; and so little is known of its history as an agent, that I am unable to date its application as a remedy earlier than 1864. If used prior to that time by the regular profession, its merits had ceased to attract attention.

I believe it was an American surgeon that first called attention to its merits in its application to indolent ulcers; after which a series of experiments were made in one or two London hospitals. So far as I know, the German and French surgeons have been silent upon the application of this simple remedy.

It not being the object of this paper to enter at length into the various trials, tests, and give the authors thereof, but simply to accept some of the conclusions drawn from them, which will afford the material for this short discussion.

The material is drawn from the periodical medical literature of the past decade, most of which has passed under your observation, and which to quote would require more time than I could spare in such a discussion.

Trials made with this remedy have all been made with dry earth. I fail to find an application reported where the clay was used even in a moist condition. I will, however, before closing, give some results from the use of medicated wet clay as a remedy under certain conditions that have not yet been reported, with which I have had some personal experience.

The kind of clay used in its dry state, I might add, the only kind is simple earth free from silicious matter.

You are aware that what we usually call clay may hold very different elements in its composition—as alumina, soda, potassa, lime, baryta, strontia, magnesia, etc. These latter are generally associated in what we style aluminous clay as distinguished from argillaceous, glaucineous and other forms of earth.

From the descriptions of the clays used, I infer that the aluminous earth is the only kind used, or that has been thus used. We find it, in this country, usually underlying the sand, or silicious rock, frequently quite pure, but more generally intermixed with silicic acid and silicates.

Since the advocates of this remedy have not indicated any special kind of clay, we conclude that it is not necessary that it should be entirely pure in order to meet the ends claimed for it. The only special directions given are, that the clay must be thoroughly dried and powdered, or at least rubbed tolerably fine before it is applied.

Under these conditions, without reference to the chemical constituency of the clay, if it exerts a uniform action upon an organized body—or rather an animal body—which does not nor can draw directly from substances so presented any nutriment, or alterative ingredient calculated to establish an organization where a disorganizing process is already set up.

This we infer from the known laws of animal growth, which is the result of food containing earthy matter taken as ingesta.

Hence the conclusion is forced upon us that any beneficial results obtained from this agent must be purely mechanical in its *modus operandi.* Leaving out of the question any direct action of dried clay—by which I mean chemical action, and which I am not prepared to positively deny—it could exert a two-fold influence:

1st. It would absorb at least twice its bulk of ichorous fluid, and at the same time exclude atmospheric air from the parts to which it should or might be applied.

It is to be regreted that the nature and character of the various ulcerations in which experiments were made with this agent, in the London infirmaries, by surgeons of known ability, were not fully described. In the reports which I have examined, they make allusions to trials in various forms of ulceration, perhaps all forms except the encephaloid, and some other forms of malignant ulcers.

If I am not mistaken, it has been tried in syphilitic nodular ulcers, with what effect memory does not serve me sufficiently to say. I will say, however, that the reports favored the remedy in all old ulcerations, claiming (perhaps too much) that at least sixty per cent. of that form of ulcers were brought to a healthy or healing condition, with the statement that those lesions which involved the muco-serous tissues, where the discharges were profuse, without rapid loss of surrounding tissue, that it proved to be the most valuable agent known to surgery.

Taking this for granted, the foregoing, without reference to any specific cause of the given case, save that, we must repeat, in the absence of proof to the contrary, that the clay acted as an absorbent of the ichorous and perhaps sanous discharges which had been acting as a foreign body in the denuded ulcer.

My first impression, on learning the beneficial results of this agent, was that the ingredients of the clay acted upon the vasculation of the parts involved; plugging up the vessels, and by excluding the air, relieved the excitation of the sentient nerves exposed, denuded, and perhaps degenerated, by the ravages of the corroding ulcers. Under this impression, I inferred that any substance that would exclude the air would answer the same purpose, provided there was no direct agency in the ingredients of the clay.

Since learning that it was only those ulcers that discharged freely of the character above alluded to, I was forced to the conclusion that the clay did not act medicinally upon the parts, but as before said, as an absorbent. Knowing its capacity for that, I abandoned the idea that simple exclusion of the atmosphere and compression was adequate to do all that was, or is, claimed for the dry clay.

I am now pretty well convinced that dry clay is a valuable adjunct in the treatment of a class of indolent ulcers; that it will remove mechanically more efficiently than any agent at our command the morbid, irritating secretions of the class of ulcers referred to, and deserves a further investigation at the hands of every honest surgeon.

Thus far we have presented the supposed efficacy of dry clay. The conditions indicating its use would be injured by its application in a wet state—styled in our language mud—which would have no absorbent properties.

It is known that wet clay in the process of drying contracts. Would we not rationally infer that it might be employed in certain forms of varicose? For instance, let the varicosed limb be enveloped, including its entire extremity, in tough clay, wetted to the consistency of dough or putty; then as soon as it is thoroughly dry, remove it, and re-apply in the same manner before the vessels has time to refill. This could be easily done, provided the hairs had been removed by close shaving.

I have seen cases resembling felon completely relieved by the application of clay in this manner, wet with strong ambier from tobacco, in which nicotine was the active agent. Perhaps atropine would have answered still better. I attribute the good effects in these cases to the uniform pressure exerted by the clay in drying, which we can obtain in no other way as effectually.

---

Onychia Maligna.—Rest and attention to the state of general health having preceded, the fungous growth is then burnt with strong nitric acid, washed with water and poulticed. The relief is certain, and the repetition of the application seldom necessary. If there should be any trouble with the nail, the tender flesh may be protected by the insertion of a thin piece of compressed sponge, kept in its place by strips of plaster applied longitudinally to avoid compression.—*British Medical Journal.*

